# Statistical modelling of transcript profiles of differentially regulated genes

**DOI:** 10.1186/1471-2199-9-66

**Published:** 2008-07-23

**Authors:** Daniel C Eastwood, Andrew Mead, Martin J Sergeant, Kerry S Burton

**Affiliations:** 1 Warwick HRI, University of Warwick, Wellesbourne, Warwickshire, CV35 9EF, UK

## Abstract

**Background:**

The vast quantities of gene expression profiling data produced in microarray studies, and the more precise quantitative PCR, are often not statistically analysed to their full potential. Previous studies have summarised gene expression profiles using simple descriptive statistics, basic analysis of variance (ANOVA) and the clustering of genes based on simple models fitted to their expression profiles over time. We report the novel application of statistical non-linear regression modelling techniques to describe the shapes of expression profiles for the fungus *Agaricus bisporus*, quantified by PCR, and for *E. coli *and *Rattus norvegicus*, using microarray technology. The use of parametric non-linear regression models provides a more precise description of expression profiles, reducing the "noise" of the raw data to produce a clear "signal" given by the fitted curve, and describing each profile with a small number of biologically interpretable parameters. This approach then allows the direct comparison and clustering of the shapes of response patterns between genes and potentially enables a greater exploration and interpretation of the biological processes driving gene expression.

**Results:**

Quantitative reverse transcriptase PCR-derived time-course data of genes were modelled. "Split-line" or "broken-stick" regression identified the initial time of gene up-regulation, enabling the classification of genes into those with primary and secondary responses. Five-day profiles were modelled using the biologically-oriented, critical exponential curve, y(t) = A + (B + Ct)R^t ^+ ε. This non-linear regression approach allowed the expression patterns for different genes to be compared in terms of curve shape, time of maximal transcript level and the decline and asymptotic response levels. Three distinct regulatory patterns were identified for the five genes studied. Applying the regression modelling approach to microarray-derived time course data allowed 11% of the *Escherichia coli *features to be fitted by an exponential function, and 25% of the *Rattus norvegicus *features could be described by the critical exponential model, all with statistical significance of p < 0.05.

**Conclusion:**

The statistical non-linear regression approaches presented in this study provide detailed biologically oriented descriptions of individual gene expression profiles, using biologically variable data to generate a set of defining parameters. These approaches have application to the modelling and greater interpretation of profiles obtained across a wide range of platforms, such as microarrays. Through careful choice of appropriate model forms, such statistical regression approaches allow an improved comparison of gene expression profiles, and may provide an approach for the greater understanding of common regulatory mechanisms between genes.

## Background

Various statistical approaches have been specifically developed to summarise the vast quantities of data that are produced in microarray studies [1-3], employing analysis of variance (ANOVA), clustering and network modelling. Analysis of variance (ANOVA) has been used to identify those gene expression responses that are most affected by different treatments, often taking account of particular forms of treatment structure, such as the correlations between sample times in a time-course study [4]. Approaches for clustering genes with similar responses range from simple methods for observed data, the calculation of correlations between genes [5], through to clustering based on linear [6] or polynomial regression [7] or spline models [8]. Network models are used to reconstruct transcription factor activity [9] or infer regulatory networks [10], assuming a particular mechanistic model for the behaviour of each regulation function based on observed microarray gene expression data.

This paper aims to use standard statistical non-linear regression models to enhance the biological interpretation of individual gene expression profiles. Such regression models provide accessible methods to describe the shape of each gene expression profile as a function of time, thus providing an insight into the underlying processes rather than simply identifying significant differences. For example, non-linear models can be used to identify the time of a particular event in a gene expression profile, such as the time of rapid up- or down-regulation. Similarly, modelling transcript changes using parametric equations that allow biological interpretation can further allow the comparison or clustering of the shapes of the expression profiles based on biological interpretable parameters. Such non-linear regression techniques are commonly used in agronomic studies to describe responses to a range of quantitative input variables, but are not commonly used in the examination of gene expression data.

The initial model system used to investigate the potential of statistical parametric, non-linear regression approaches for gene profiling was fungal morphogenesis with data provided by quantitative reverse transcriptase PCR (qRT-PCR) which provides a more precise method than either Northern analysis or microarrays [11]. This system encompasses a range of growth forms from vegetative mycelium to multicellular organs which enable the fungus to respond to changes in nutrition and environment, and undergo pathogenesis or reproduction. The fruiting bodies of the basidiomycete fungus *Agaricus bisporus*, the cultivated mushroom, are ideal for studying fungal morphogenesis as they are macroscopic, the tissues are clearly de-lineated (stipe, caps and gills) and the initiation of fruiting body morphogenesis is controlled environmentally. Differential screening and targeted gene cloning procedures have identified genes up-regulated post-harvest in *A. bisporus *fruiting bodies, based on Northern analysis [12-14]. Genes have also been identified which are expressed in developing fruiting bodies of several other fungi, including *Lentinula edodes *[15], *Pleurotus ostreatus *[16], *Flammulina velutipes *[17] and *Coprinus cinereus *(*Coprinopsis cinerea*) [18,19].

This study investigated how expression profiles, generated from qRT-PCR data of differentially regulated genes in *A. bisporus *fruiting bodies, could be statistically modelled both to estimate the time of up-regulation and determine similar temporal expression patterns. The five genes chosen for profiling are functionally distinct, and therefore unlikely to have obvious common regulatory mechanisms: they are cruciform DNA binding protein, cytochrome P450II, β (1–6) glucan synthase, glucuronyl hydrolase and riboflavin aldehyde-forming enzyme [12,20]. Whilst being functionally distinct, these genes were expected to show broadly similar patterns of expression following harvest, allowing the fitting of a single model form to all five responses. This enables the comparison of the profiles via biologically interpretable parameters rather than simply clustering genes based on the observed data. To determine the time when transcription first increased for each gene, transcript levels of each gene were examined at 3 h intervals for 24 h. Transcript levels were also measured at 24 hour intervals over 5 days. These profiles were modelled to provide directly interpretable parameter values, offering an insight into the regulation of the genes. Spatial control of gene expression was also assessed by comparing transcription in tissues of the harvested mushroom during the first 48 hours post-harvest storage.

Furthermore, this study has applied these regression modelling approaches to publicly-available microarray data sets from published studies, to identify groups of genes showing similar regulatory patterns. The potential application of this approach to fully exploit the large quantities of high-throughout data was discussed.

## Results

The qRT-PCR-derived data were initially assessed to ensure suitability for further study. Standard curves show that the PCR reactions were operating at close to 100% efficiency. Melting curve analyses showed that in all cases a single product was obtained showing that the specificity of the reaction was high. The PCR control treatments showed no evidence of DNA contamination or active reverse transcription after the 85°C treatment (data not shown).

### Comparison of methods of measuring gene expression in *A. bisporus*

**Figure 1:**
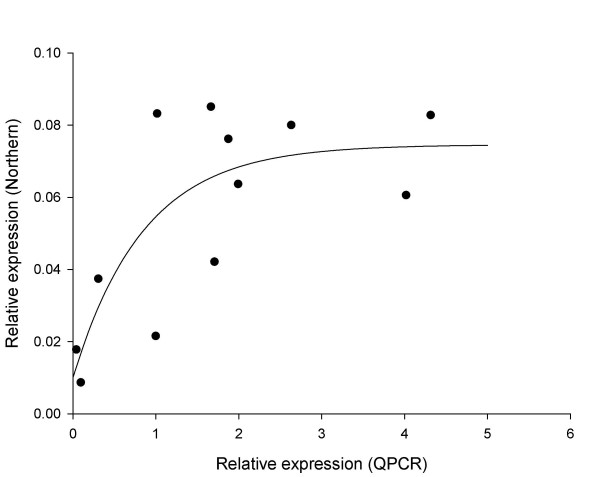
**Example showing the saturation of Northern-derived signal**. Exponential regression curve showing the relationship between the Northern analysis response (y axis) and the qRT-PCR response (x axis) for glucuronyl hydrolase during 5 days post-harvest storage. Fitted equation is y = 0.0746 – 0.0646 * 0.309^x^, R^2 ^= 52.5%.

Gene transcript levels were obtained by qRT-PCR and Northern analysis (see Additional file 1) from the same RNA extracts obtained from the mushroom tissues. These data were then compared by calculating correlation coefficients and fitting simple linear regression relationships for Northern analysis response as a function of the qRT-PCR response, for each gene separately within each of the three experiments. Northern analysis responses increased with increasing values of the qRT-PCR response, and in most cases linear regression lines fitted well (see Additional file 2). However, the Northern analysis response appears to reach an upper threshold at high values of the qRT-PCR response, e.g. for cruciform DNA-binding enzyme and glucuronyl hydrolase (0–5d and tissues experiments), and β(1–6) glucan synthase (tissues experiment). Exponential regression curves were then fitted to the responses and for ten of the 15 data sets these curves provided a better fit than the simple linear regression models (see Additional file 3), with the curvature of the response suggesting asymptotic regression, for example, glucuronyl hydrolase, 0–5d experiment (Figure 1).

### Transcription in the first 24 hours (*A. bisporus*)

**Figure 2:**
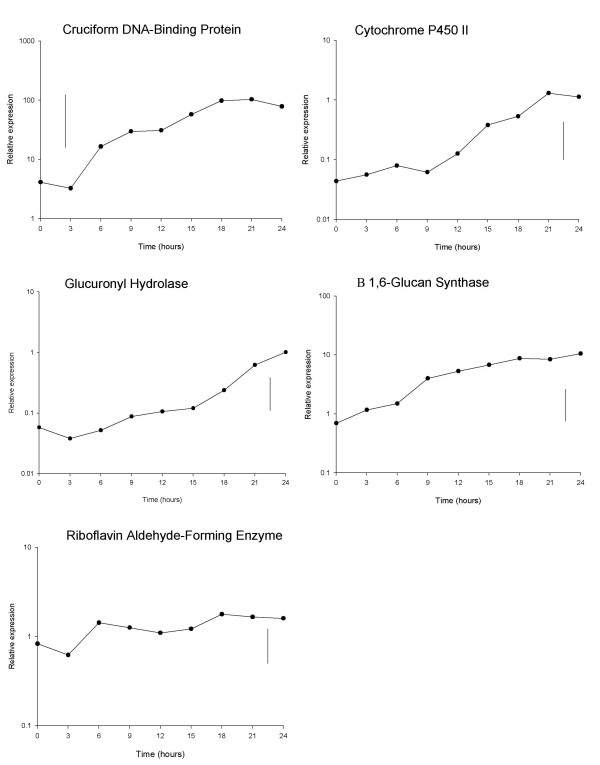
**Gene expression during the first 24 hours post-harvest sampling at 3 hourly intervals**. Mean qRT-PCR (log_10_-transformed) measurements of gene expression of the 5 selected genes during the first 24 hours post-harvest storage, sampling at 3 hourly intervals. Vertical lines show LSD (5%, 16d.f.) for comparing means at different times, as calculated from the residual mean square obtained from the ANOVA.

The different expression patterns for each gene over the first 24 hours following harvest, as obtained using qRT-PCR, are shown in Figure 2. During this period, transcript levels increased significantly, by approximately 10 fold or more, for all genes except riboflavin aldehyde-forming enzyme (less than 2 fold). Transcript levels of cruciform DNA-binding protein increased shortly after harvest to very high levels, while for cytochrome P450II and glucuronyl hydrolase the profiles showed an initial period of little change followed by increases in transcript levels (approx. 12 and 18 h after harvest respectively). In contrast the transcript levels of β(1–6) glucan synthase increased continuously during the first 24 hours post-harvest, with a notable sudden increase at about 9 h.

**Figure 3:**
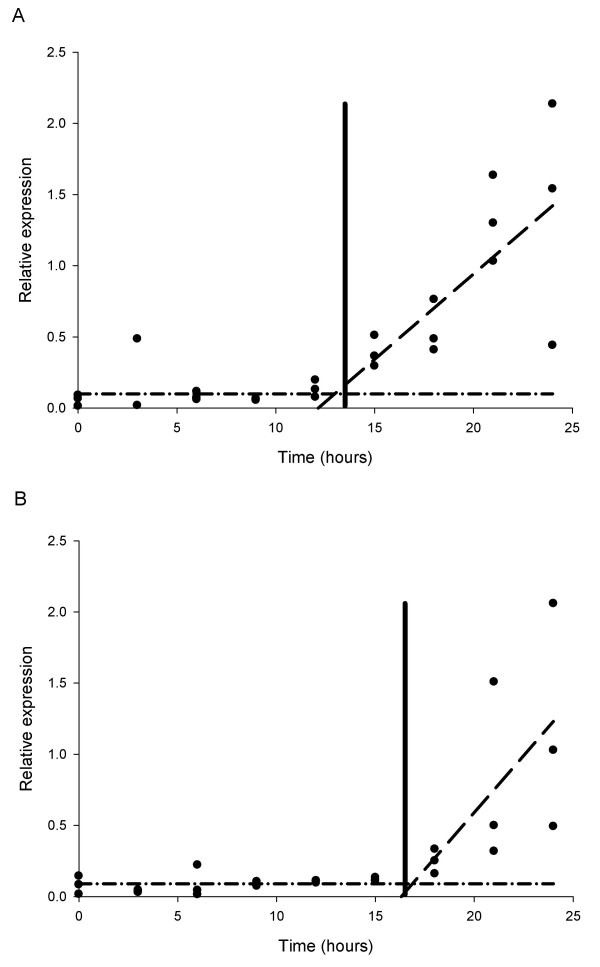
**Broken-stick analysis showing the point of increased transcription**. A) cytochrome P450II (CYPII) at 13.5 hours (+/- 2.13 hours) and B) glucuronyl hydrolase (GHYD) at 16.5 hours (+/- 2.05 hours) in *A. bisporus *fruiting bodies over the first 24 hours following harvest, sampling at 3 hourly intervals.

'Split-line' or 'broken-stick' analysis was used to model the transcription profile data from the first 24 hours to estimate the time at which an initial increase in transcription occurred. The model was applied successfully to the transcript data for cytochrome P450II and glucuronyl hydrolase, with these data sets each described by two separate linear regression line segments, the first line having a slope of zero (Figure 3). More precise estimates of when transcription first increased were determined in these analysis as 13.5 hours (+/- 2.1 hours (95% confidence interval)) for cytochrome P450II (Figure 3A) and 16.5 hours (+/- 2.1 hours (95% confidence interval)) for glucuronyl hydrolase (Figure 3B). This analytical approach was not successful in describing the transcription profiles of cruciform DNA-binding protein and β(1–6) glucan synthase, as there were insufficient early time points to establish an initial baseline response (line with zero slope). Transcripts of riboflavin aldehyde-forming enzyme showed no clear upward trend in the first 24 hours, and so the 'broken-stick' analysis approach was again not successful.

### Transcription profiling over 5 days post-harvest (*A. bisporus*)

**Figure 4:**
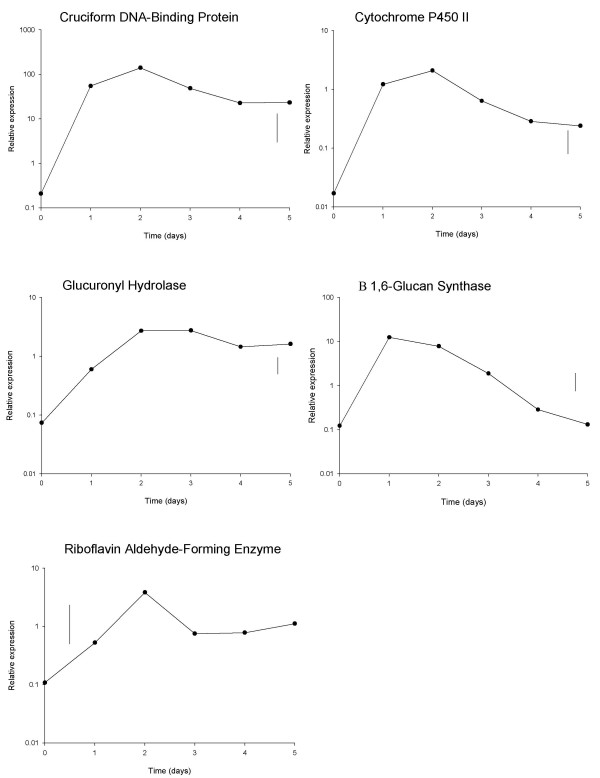
**Gene expression over 5 days post-harvest development**. Mean qRT-PCR (log_10_-transformed) measurements of gene expression of the 5 selected genes during 5 days post-harvest storage, sampling at 24 hour intervals. Vertical lines show LSD (5%, 10d.f.) for comparing means at different times, as calculated from the residual mean square obtained from the ANOVA.

The 5-day transcript profiles, as obtained using qRT-PCR, showed an initial increase in transcript levels for all five genes for 1–2 days, followed by a plateau or a decline (Figure 4). The extent of increase in transcript levels over the first two days ranged from 668 fold for cruciform DNA-binding protein, to 47 fold for riboflavin aldehyde-forming enzyme and 36 fold for glucuronyl hydrolase. Gene expression over five days storage, as measured using qRT-PCR, was modelled as a function of storage time by non-linear regression analysis of log_10_-transformed data using a critical exponential curve (Equation 1: y(t) = A + (B + Ct)R^t ^+ ε). This curve was selected in preference over either the simpler single exponential curve or more complex double exponential curve, based both on the shape of the response, and a formal comparison of the goodness of fit of each model. This was achieved by comparing each more complex model to the simpler alternative (the critical exponential can be considered a simpler alternative to the double exponential, and the single exponential a simpler alternative to the critical exponential). The improvement in fit was assessed using an F-test to identify the significance of the ratio of the change in residual variance between models to the residual variance for the more complex model.

For all genes, there was a significant improvement in the fit when choosing the critical exponential rather than the single exponential, but no significant improvement when choosing the double exponential over the critical exponential (data not shown). Simultaneous fitting of the critical exponential curve to the data for all five genes allowed a comparison of the fitted parameters, and hence the detection of degrees of commonality between the patterns of expression for the genes. This analysis identified that there was no significant improvement to the fit when allowing parameter *R *(related to the curvature of the response) to be different for the five genes, and so this parameter could be constrained to be the same. However, constraining the other three parameters to be the same across all five genes resulted in significantly worse fits, though for some pairs of genes the fitted (and derived) parameters did suggest some similarities (Table 1). The different parameters (both fitted and derived) can be interpreted in terms of particular features of the shape of the response. The size of parameter *C *indicates the magnitude of the decline from the maximum expression response, whilst the ratio of *B *over *C *is related to the time to the maximum response. Parameter *A *measures the asymptotic response level after lengthy storage, *A *+ *B *is the response at time t = 0, and the maximum response is dependent on all four parameters, obtained by inserting the time of maximum response into the critical exponential equation (Equation 1).

**Figure 5:**
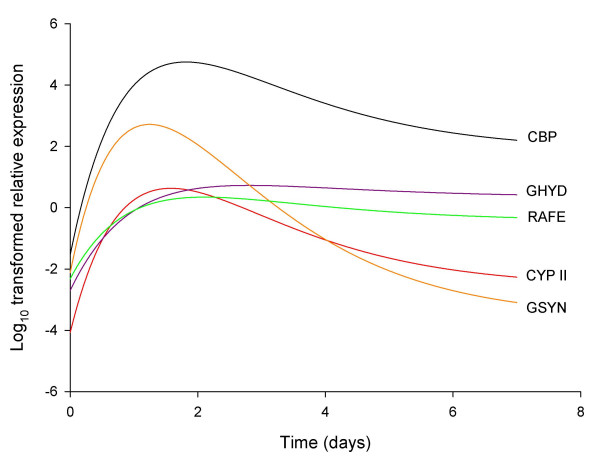
**Critical exponential curve models for transcription patterns during 5 days post-harvest development**. Sampling was conducted at 24 hour intervals. Models for log_10_-transformed qRT-PCR gene expression as given by Equation 1, with fitted parameters as given in Table 1. CBP (black line) = cruciform DNA-binding protein, CYPII (red line) = cytochrome P450II, GHYD (blue line) = glucuronyl hydrolase, GSYN (orange line) = β (1–6) glucan synthase, and RAFE (green line) = riboflavin aldehyde-forming enzyme.

The analysis identified three distinct regulatory patterns (Figure 5). Cruciform DNA-binding protein and cytochrome P450II were identified as having similar shaped curves (similar values of *C*) and similar times of peak transcript levels (similar ratios of *B *over *C*), despite large differences in transcript magnitudes during this five-day period (different values of both *A *and *A*+*B*). Similarly, the transcript profiles of glucuronyl hydrolase and riboflavin aldehyde-forming enzyme shared a common curve shape (similar values of *C*) and similar transcript values for both the initial and asymptotic parts of the curve (similar values of both A and *A*+*B*). The time of maximum transcript level of each of these genes was later than for the other genes examined (larger ratios of *B *over *C*). The 5 day β(1–6) glucan synthase profile had a different curve shape with the earliest peak of transcript level (smallest ratio of *B *over *C*) followed by a rapid decline (largest value of *C*, smallest value of *A*).

**Table 1:** Critical exponential curve models for the transcription patterns of the 5 genes during long-term *A. bisporus *fruitbody post-harvest storage.

	Fitted parameters			Derived descriptors			
		
	A	B	C	A+B	-B/C	Time of maximum response (days)	Maximum response
CBP	1.886	-3.426	7.801	-1.540	0.439	1.814	1.753
CYP II	-2.572	-1.500	7.350	-4.072	0.204	1.579	0.633
GHYD	0.348	-3.049	2.125	-2.701	1.435	2.810	0.727
GSYN	-3.592	1.473	11.350	-2.119	-0.130	1.245	2.717
RAFE	-0.423	-1.896	2.585	-2.319	0.733	2.108	0.344

Fitted parameters for five genes (CBP = cruciform DNA-binding protein, CYP II = cytochrome P450II, GHYD = glucuronyl hydrolase, GSYN = β (1–6) glucan synthase, and RAFE = riboflavin aldehyde-forming enzyme) with parameter R constrained to be common across all five genes (R = 0.4832 (s.e. = 0.0288)). Additional descriptors of response shape derived from the fitted parameters.

### Transcript expression between tissues (*A. bisporus*)

**Figure 6:**
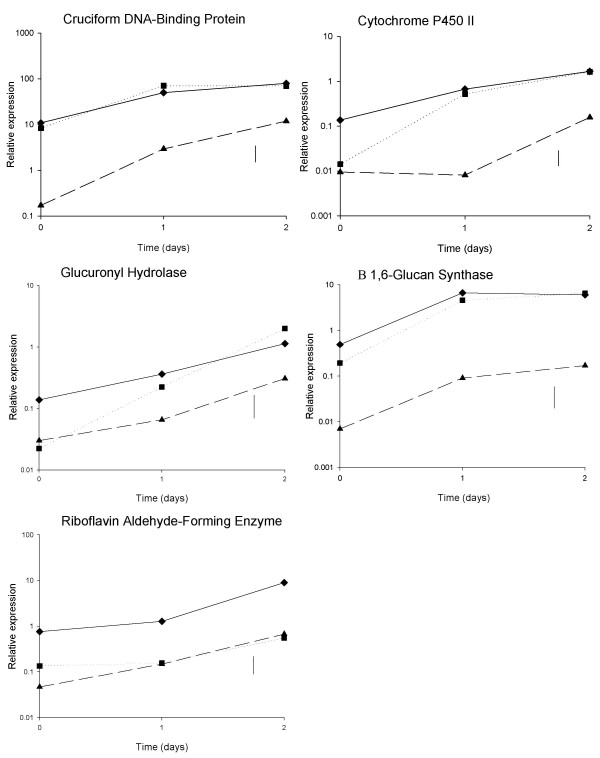
**Gene expression in stipe, cap and gill tissues post-harvest**. Mean qRT-PCR (log_10_-transformed) measurements of gene expression of the 5 selected genes at 24 hour intervals post-harvest for 2 days, where mushrooms were dissected into stipe (◆), cap (■) and gill (▲) tissue. Vertical lines show LSD (5%, 16d.f.) for comparing means for different combinations of time and tissue, as calculated from the residual mean square obtained from the ANOVA.

Transcript levels of each gene were measured in stipe, cap and gill tissues of harvested mushrooms over 2 days storage, using qRT-PCR (Figure 6). Differences in transcript levels, between tissues, between storage times and due to the interaction of these two factors, were assessed using ANOVA. Average transcript levels of cruciform DNA-binding protein, cytochrome P450II, glucuronyl hydrolase and β (1–6) glucan synthase in stipe and cap tissues were similar and significantly higher than in the gills. For riboflavin aldehyde-forming enzyme, transcript levels were significantly higher in the stipe tissue compared with the cap or gills, which had similar levels. Despite differences in transcript levels observed between the tissues, all genes showed an increased level of expression in all tissues from day 0 to 2.

### Application of regression approaches to microarray datasets (*E. coli *and *R. norvegicus*)

**Figure 7:**
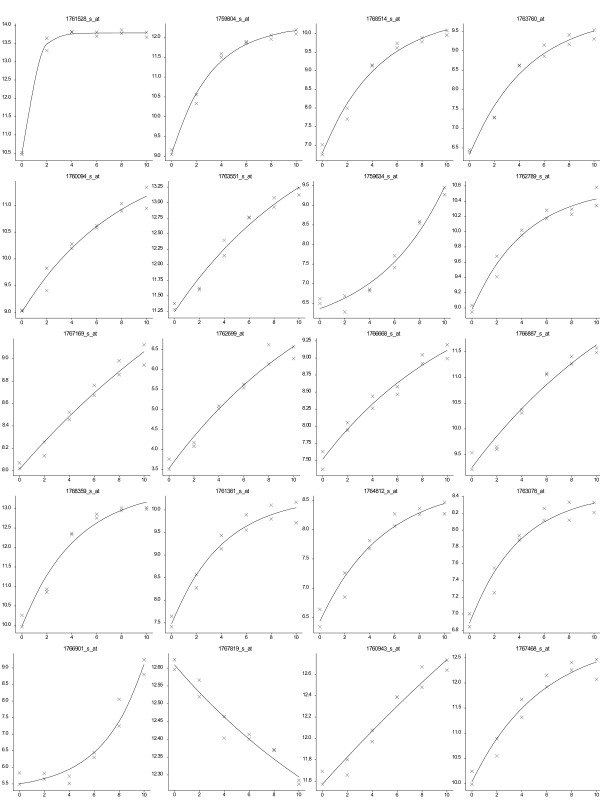
**Gene expression profiles from *E. coli *microarray study**. Fitted exponential curves for the 20 best fitting profiles together with observed data. Horizontal axis is time in minutes, vertical axis is log (base 2) expression value.

Regression analyses were applied to time course profiles obtained from two published microarray datasets [21,22]. Gene responses of *E. coli *to treatment with paraquat [21] were analysed by fitting an exponential function. From over 10,000 features on the microarray the profiles for 11% were described well by the exponential function, with significance levels of p < 0.05; 2% of the gene profiles fitted with significance levels of p < 0.001 (Figure 7).

**Figure 8:**
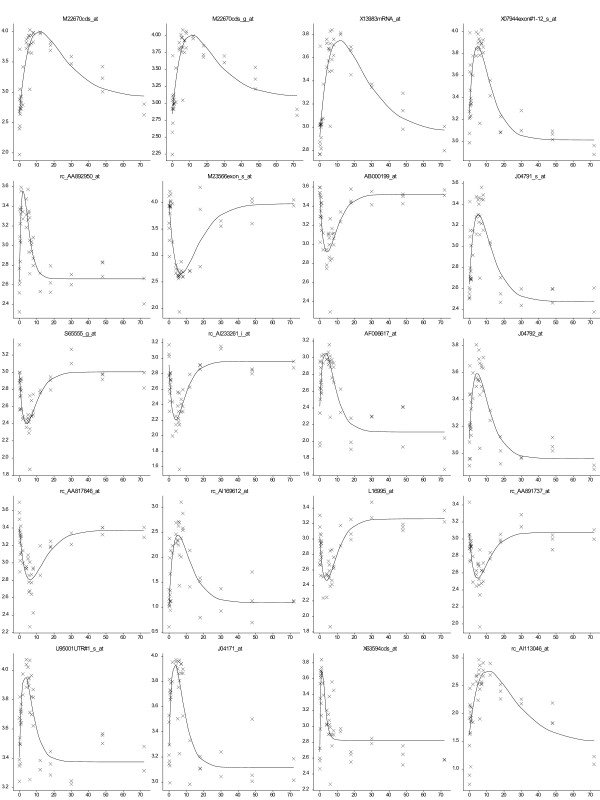
**Gene expression profiles from *Rattus norvegicus *microarray study**. Fitted critical exponential curves for the 20 best fitting profiles together with observed data. Horizontal axis is time in hours, vertical axis is log (base 10) expression value.

The critical exponential function was fitted to the gene time-course responses in the rat liver tissue to the corticosteroid, methylprednisolone, [22]. The *R. norvegicus *microarray (Affymetrix GeneChips Rat Genome U34A) consisted of 7000 full length sequences and over 1000 expressed sequence tagged clusters. From over 8000 features on the microarray, the profiles for over 25% were described well by the critical exponential (p < 0.05), with over 9% with significant levels of p < 0.001 (Figure 8).

For both studies the chosen functions allow the description of a number of distinct forms of response. The fitted responses for the 20 most significant fits from each study demonstrate the variety of profiles that can be described by each of these models (Figures 7 and 8).

## Discussion

Gene expression studies were conducted to investigate the benefit of applying statistical regression approaches to the fitting of mathematical models for the analysis of transcriptional data. In this study these approaches have been developed using precisely measured transcript levels for a small number of genes and application of the approaches has been further demonstrated for microarray datasets. As microarray technologies continue to be developed, the variability of gene expression data from such technologies will be reduced, leading to the widespread application of the regression modelling techniques developed in this study, thus allowing the comparative analysis of larger numbers of gene expression profiles. A range of statistical regression techniques, both linear and non-linear, are readily available. The selection of appropriate techniques is critically dependent on the specific question being addressed and the data that are collected.

For *A. bisporus*, the increases in transcript levels in the first 24 hours following harvest are largely due to transcription rather than losses due to mRNA turnover. 'Split-line or 'broken-stick' regression analysis was used to calculate the time when transcription was initiated, at 13.5 h post-harvest for cytochrome P450II and 16.5 h post-harvest for glucuronyl hydrolase. The novel application of this approach to transcript profiling demonstrates the potential value of this simple mathematical model which has been used previously in such diverse applications as estimating the thresholds for patch size in ecological studies [23], humidity levels in plant pathogen germination experiments [24] and the mineral density of bones [25]. Successful application of the 'split-line' or 'broken-stick' model for cruciform DNA-binding protein and β (1–6) glucan synthase would require more sampling points to be made in the first 6 hours to provide sufficient time points to allow the fitting of the baseline response, zero-slope line, and hence allow estimation of the time of initial transcription. For the riboflavin aldehyde-forming enzyme further data are needed beyond 24 hours (but still at 3-hour intervals) to allow estimation of the second linear regression segment, and to again allow calculation of the time of initial transcription.

The time of increased transcription for at least 4 of the 5 tested genes occurs at different times in the first 24 hours post-harvest. This suggests that the response of the mushroom to harvest is not under the control of a single regulatory pathway, such as the signal transduction pathways described for fungal oxidative and osmotic stress responses [26]. The controlling events in the mushroom are likely to be affected by a range of stimuli, such as stress, nutrient limitation, continued maturation and spore formation, which might illicit both primary or immediate responses, and secondary responses. Here we observed increased transcription first of cruciform DNA-binding protein (approximately 3–6 h), followed by β (1–6) glucan synthase (approximately 9 h), cytochrome P450II (13.5 h), glucuronyl hydrolase (16.5 h) and riboflavin aldehyde-forming enzyme (> 24 h). Cruciform DNA-binding protein and β (1–6) glucan synthase may be part of a primary response, while cytochrome P450II, glucuronyl hydrolase and riboflavin aldehyde-forming enzyme are from a secondary response or caused by a later stimulus.

Statistical regression modelling showed similar expression patterns for two *A. bisporus *genes, glucuronyl hydrolase and riboflavin aldehyde-forming enzyme, the latter of which is known to be up-regulated during the development of the non-harvested mushroom [20]. Further study is required to determine whether the common pattern observed between the genes post-harvest is also observed in the morphogenesis of the non-harvested fruiting body. The pattern of β (1–6) glucan synthase gene expression during 5 days storage was different from the other genes studied, with transcript levels falling greatly after 2 d following an initial increase in gene expression. The gene has been hypothesised to be involved in cell wall synthesis [12]. The initial increase in gene expression coincides with the period when hyphae in the cap and stipe elongate, i.e. in the first 2 days post-harvest, a process for which cell wall synthesis is important. Similarly, the reduction in expression after day 2 coincides with the cessation of cell wall synthesis following the full extension of the cap [27,28].

The fitted critical exponential models for the 5-day profiles of cruciform DNA-binding protein and cytochrome P450II had similar shapes, whilst the times of initial transcriptional increase for these two genes, as determined by split-line regressions, were markedly different (3–6 h and 13.5 h respectively). This apparent paradox illustrates the importance of considering transcript responses over a number of different time-scales. The precise function of cruciform DNA-binding protein is not known, however, it is unlikely to be involved in recombination as transcript levels are low in the gill tissue where meiosis occurs. In other organisms, proteins with similar cruciform DNA-binding activity (i.e. HMG proteins) cause the increased and decreased transcription of genes [29,30]. It is possible that the early and abundant transcription of cruciform DNA-binding protein in *A. bisporus *fruiting bodies acts to regulate the expression of other genes.

The spatial transcript levels were different between tissues and for all genes were significantly lower in the gill tissue, indicating a physiological difference from the stipe and cap. The gills are actively growing, respiring and producing spores via meiosis. They are a nutrient 'sink' and are therefore physiologically different from the stipe and cap tissues, which export nutrients and are subject to stress from cell damage. However the increased respiration in gill tissue [31,28] may result in increased ribosomal, and therefore 18S rRNA, levels, to which transcript levels from qRT-PCR are normalised, so the quantitative differences between gills and stipe/cap tissues may be influenced by this. While pairs of genes showed similar patterns of response when described using the critical exponential model, the spatial distribution of transcripts between different tissues varied in some cases. For example, cytochrome P450II and cruciform DNA-binding protein showed similar overall patterns, but cytochrome P450II showed a delayed initial increase in transcription in the gill tissue. Further and more detailed studies of the expression of these genes in the separate tissues are needed to understand the potentially different regulatory mechanisms acting in each tissue.

The use of a statistical non-linear regression approach to model the gene expression profiles over an extended period offers the opportunity to compare the shapes of the response curves between genes. The critical exponential curve, selected by the observed response shapes and goodness of fit, can be explained in terms of the combination of two processes, in this case RNA synthesis and degradation. A more complex model for such a situation would be the double exponential curve, which is a natural function for a two-timescale process. The critical exponential is the degenerate case of this model and occurs when two processes in a system have the same timescale. In this case, however, there was no evidence for choosing the more complex model. Choice of an appropriate model can be important, but many common non-linear models are based on functional forms derived from observed biological processes. Increased gene transcription is responsible for the initial rapid increase in transcript levels between days 0 to 2. Transcript levels continue to rise until a maximum is reached, followed by a decline towards a steady level, possibly as a consequence of a balance between transcription and degradation (transcript turnover).

The critical exponential model was successful in identifying genes with quantitatively similar patterns of response, which could not have been predicted from their putative protein functions. This approach, therefore, offers a new method by which a large number of genes could be classified according to their initial transcription regulation and subsequent turnover.

Our approach to first model and then cluster allows more genes to be considered and potentially a greater insight to understand the system. The application of this approach to a microarray dataset allows the screening of genes to identify responses that can be described by a particular mathematical function. Thus each gene profile is reduced from noisy observations to a smaller set of biologically-interpretable parameters. In the analysis of the microarray datasets, different shapes of profiles fitted by the exponential or critical exponential functions can be identified (Figures 7 &8), allowing the grouping of genes based on the parameter values. Eliminating the inherent variability in the data through the regression modelling approach allows a more precise comparison of gene profiles and thus improved clustering. For example, the 9% of *R. norvegicus *genes identified that followed the critical exponential curve at the p < 0.001 level represents approx 720 genes compared with the 200 genes identified and modelled by Jin *et al *(2003) [22]. The groupings of genes then generated by the improved clustering propose hypotheses of regulatory association between genes. For example, the aim of the *E. coli *microarray study was to identify those genes co-regulated with the main regulatory gene, *sox*S, which was demonstrated to have an exponential-type response following the application of paraquat [21]. By using an initial regression analysis to identify the subset of genes that can be described by an exponential function, subsequent cluster analyses can focus on this subset of genes with similar, but not identical (see Figure 7), shapes of expression profiles. The fitted exponential function parameters for this gene subset could then be used to better identify those genes most closely co-regulated with *sox*S. Whilst our study demonstrates the application of a regression modelling approach to describe gene expression profiles, this approach can be expanded to fully exploit microarray datasets. Application of a wider range of functional forms (for example including functions with similar shapes but also some temporal variation or time delays) offers the potential to develop regulatory networks based on relationships between the shapes of expression profiles as captured by the fitted parameters. Further interpretation of the parameters, alongside knowledge of gene function, might allow the identification of the stimuli driving the observed gene expression responses.

Compared with standard clustering for gene profiles of microarray data, the statistical regression modelling of mathematical functions to describe these profiles eliminates the inherent variability in the data and allows the direct comparison of profile shapes.

## Conclusion

This study has illustrated how the use of standard statistical modelling approaches (analysis of variances (ANOVA), linear regression modelling, non-linear regression modelling) commonly used in plant, microbial and ecological sciences, can be used to aid and extend the interpretation of gene expression profiles obtained from qRT-PCR. These approaches have been applied to model profiles of larger numbers of genes obtained from expression microarray studies, and could be further applied to other high-throughput "-omic" technologies. A wide range of statistical approaches have been specifically developed to analyse the vast quantities of data generated in microarray-based studies [32], assessing both similarities and differences between genes and between treatments. Similarly a number of approaches have been developed to generate mathematical models for assumed networks of gene pathways (based on simple mathematical assumptions) [33,34]. However, there appears to have been little statistical consideration for the detailed modelling of individual gene expression profiles. The statistical regression modelling approaches applied in this study allow the estimation of parameters which succinctly describe the shapes of gene expression profiles. These parameter estimates (or combinations of them) can then be related directly to the processes stimulating and driving the expression of these genes. Comparison of the parameters and expression profiles for a set of genes could then indicate that a sub-set of these genes are co-regulated, with the potential to hypothesise a common regulatory mechanism. Hence, consideration of a wide range of non-linear regression models could provide building blocks for the development of more biologically realistic models of gene expression profiles.

## Methods

*Agaricus bisporus *strain A15 (Sylvan, UK) was used throughout the study. Mushrooms were grown on composted wheat straw according to commercial practice at the Warwick HRI BioConversion Unit. Mushrooms were harvested at morphogenetic stage 2 [31] and were either frozen immediately under liquid nitrogen, termed time 0, or stored for a specified period in a controlled environment, 18°C and 95–95% relative humidity, before freezing under liquid nitrogen. Stored mushrooms were sampled for gene expression profiling over i) 0 to 24 hour time course post-harvest (three hourly intervals), ii) 0 to 5 day time course following harvest (24 hourly intervals), and iii) 0 to 48 hours post-harvest (24 hour intervals), with mushrooms dissected into stipe, cap and gill tissues. Three replicate mushrooms were taken for each sampling point and frozen samples were stored at -80°C.

### RNA isolation

RNA was isolated from mushroom tissues according to established phenol/chloroform extraction protocols [35]. Absorbance measurement at 260 nm and 280 nm were used to assess RNA concentration and purity. RNA integrity was determined with formaldehyde agarose gel electrophoresis [36]. For experiments involving reverse transcriptase, RNA samples were treated with RQ1 RNAse-free DNAse enzyme (Promega, Southampton, UK) according to manufacturer's instructions

### Quantitative RT-PCR (qRT-PCR)

Transcript levels were determined using the ABI Prism 7900 HT sequence detector (TaqMan™) and SYBR^® ^Green fluorescent reporter dye. Reverse transcription was carried out using the Thermoscript™ RT-PCR system (Invitrogen, Life Technologies, Paisley, UK) in 20 μl volumes containing 50 ngμl^-1 ^random hexamers, 1 μg total RNA, 1 μl Thermoscript reverse transcriptase (15 U μl^-1^), 4 μl 5× Thermoscript™ buffer, 1 μl 0.1 M DTT and 1 μl RNaseOUT™ (40 U μl^-1^). Reactions were carried out at 25°C for 10 minutes, followed by 50 minutes at 50°C and terminated at 85°C for 5 minutes. Each cDNA sample was treated with RNAse H according to manufacturer's instructions and diluted to 100 μl final volume. cDNA samples were taken from three replicate mushrooms per time point.

PCR reactions were performed in 15 μl volumes consisting of 1 μM of each primer, 20 ng of cDNA sample and 7.5 μl of 2× SYBR^® ^Green PCR Mix (Applied Biosystems, Warrington, UK). All sample and standard reactions were carried out in triplicate. PCR cycling conditions consisted of one cycle of 50°C for 2 min and 95°C for 10 min followed by 40 cycles 95°C 15 sec and 60°C for 1 min. This was followed by a dissociation step of 95°C for 15 sec, 60°C for 15 sec and increase to 95°C with a 2% ramp rate. The dissociation step was used for melting curve analysis in order to detect primer dimers and non-specific products in the reaction. Standard curves were generated for each gene using cDNA (0.625 ngμl^-1 ^to 20 ngμl^-1^) from a whole 2-day stored mushroom, which contains large amounts of target transcripts. The primer sets used (Table 2) were designed from cDNA sequence information for each gene [12] using the Primer Express^® ^software version 2.0 Applied Biosystems, Warrington, UK).

**Table 2:** Oligonucleotides used in the qPCR for the selected *Agaricus bisporus *genes

**Gene**	**Oligonucleotide**	**Amplicon ** **length (bp)**
**Cruciform DNA-binding protein**	Forward: 5'-CGCTGGTGAAGCTGAGAACA-3'	74
	Reverse: 5'- CAGCGATTTGGTCCGTCATA-3'	

**Cytochrome P450II**	Forward: 5'-GCCGATATTTTGCTCTGAATGC-3'	75
	Reverse: 5'- GCGCAGGCTTGATATCGAA-3'	

β **(1–6) Glucan synthase**	Forward: 5'-TCAATCTTCTTGATGCTCATTGC-3'	70
	Reverse: 5'- TGCGCAAACAACCTATTCC-3'	

**Glucuronyl hydrolase**	Forward: 5'-TGATGGAATTGTACCATGGGATT-3'	74
	Reverse: 5'- AGCGATAGTTGCTGCTGAAGAA-3'	

**Riboflavin aldehyde-forming enzyme**	Forward: 5'-CGGCAGCGGAGACCATT-3'	65
	Reverse: 5'- TGACTTTCACGTATTTGCTTTGT-3'	

**18S rRNA**	Forward: 5'-ACAACGAGACCTTAACCTGCTAA-3'	78
	Reverse: 5'- GACGCTGACAGTCCCTCTAAGAA-3'	

Data analysis utilised the ABI PRISM sequence detector^® ^software (SDS) (version 2.0) to determine the cycle threshold of each sample (Ct-Target) which was normalised to the cycle threshold of the 18S rRNA qRT-PCR product (Ct-Control) for the same sample [37,38]. The ΔCt equation (ΔCt = 2^(CtControl-CtTarget)^) was used to calculate the amount of each target transcript relative to the amount of 18S rRNA.

The control treatments were (a) water control using sterilised diethylpyrocarbonate (DEPC)-treated water in the place of the cDNA sample to detect environmental DNA contamination and primer-based artefacts (b) DNAse-treated RNA to assess for contaminating DNA in the RNA samples, and (c) the absence of primers during the reverse transcription step, but present during PCR, to detect contaminating DNA and the possibility of active reverse transcriptase present during PCR.

### Northern hybridisation analysis

Total RNA, ~10 μg, from each sample was separated by formaldehyde agarose gel electrophoresis and immobilised onto nylon membranes as per established protocols [35]. Hybridisation was carried out using randomly primed [α-^32^P]dCTP probes and post-hybridisation washes carried out using established protocols [35]. To produce the probes, phagemid clones containing the cDNAs were restricted with *Hin*dIII and *Bam*HI and fragments separated by agarose gel electrophoresis, excised and purified using the Qiagen gel purification protocol. Purified fragments were used as templates for random priming incorporating [α-^32^P]dCTP (*Redi*prime kit, Amersham Pharmacia Biotech., Buckinghamshire, UK). *Agaricus bisporus *28S rRNA gene was used as a loading control as described previously [12,39]. Hybridisation intensity of each gene-specific probe used in Northern analysis was determined using scanning densitometry (Personal Densitometer SI, Molecular Dynamics, CA, USA). Transcript levels for each gene were calculated relative to the hybridisation intensity recorded for the 28S rRNA gene probe for each sample tested. Northern analysis was performed on total RNA from two replicate mushrooms per time sample or time × tissue sample for transcripts of each gene examined.

### Statistical analysis

Comparisons of the transcript levels, as determined by Northern analysis scanning densitometry and qRT-PCR, were made for each gene by calculating correlation coefficients, and by fitting linear and exponential regression responses to explain the Northern analysis measurements in terms of those from the qRT-PCR. Within each experiment, two replicate Northern analysis measurements were paired with the qRT-PCR values obtained from the same replicate mushroom RNA extracts (note that for one replicate of each sampling point within each experiment no Northern analysis measurement was obtained).

Quantitative RT-PCR data of transcription levels for each gene were analysed using analysis of variance (ANOVA) for each experiment (0–24 h, 0–5d and 0–48 h between different tissues) separately. Three replicate mushrooms were assayed at each time or for each tissue-by-time combination. Prior to analysis, the data were subjected to a logarithm (base 10) transformation to satisfy the ANOVA assumption of homogeneity of variance. The significance of the overall treatment effects (time only in two experiments, time, tissue and the interaction between these factors in the third) was assessed using an F-test, and the significance of differences between individual treatment means was assessed by comparison with appropriate standard errors of differences (SEDs). Treatment differences noted in the text are significant at the 5% level unless stated otherwise.

For the qRT-PCR data only, regression analyses were used to model the gene expression changes over time. 'Split-line' or 'broken-stick' regression analysis of transcription levels from 0–24 h was applied to estimate the time when the up-regulation of each gene commenced. The 'broken-stick' model consists of two linear regression segments fitted to distinct subsets of the data, with separate estimates of slope and intercept for each segment. In this case the first line segment was constrained to have a slope of zero. A sequence of models was fitted to the data for each gene, splitting the data set into two parts (time ≤ *x *hours: time > *x *hours, for each of the observed values of *x*). The best model for each gene was chosen as the one with the minimum sum of residual sums of squares for the two regressions. The time point where the two lines crossed was postulated as the time when increased gene transcription began.

The long-term gene transcript profiles (0–5d) were modelled using the critical exponential curve (Equation 1), fitted to the log_10_-transformed data.

*y*(*t*) = *A *+ (*B *+ *Ct*)*R*^*t *^+ ε (*Equation *1)

where *A*, *B*, *C *and *R *are parameters, *y *is the gene expression response (log_10 _transformed), *t *is storage time, and ε represents the errors, assumed to follow a Normal distribution with mean zero and a constant variance. This form of curve was selected following an initial graphing of the responses, as it can be used to describe a rapidly increasing phase followed by a decline or plateau, and after assessment of how well it fitted the observed data compared with both the simpler exponential model and the more complex double exponential model. The parameters of this non-linear response can be interpreted in terms of a postulated mechanism driving the observed gene expression responses, in this case potentially quantifying the relationship between transcript synthesis and degradation. The fitted parameters, and hence the shapes of the fitted curves, were compared between genes using a parallel curves analysis, either constraining each parameter to be the same across all five genes, or allowing variation in the values taken by each parameter between genes. This analysis provides a basis for comparing a sequence of possible models, and assessment of the change in residual variance between models allows the most appropriate model for the observed data to be determined.

### Application of regression modelling to microarray datasets

The regression modelling approach was applied to publicly-available microarray datasets from different organisms (*E. coli *and *R. norvegicus*), previously published [21,22]. The datasets were selected as having an appropriate time course with a fixed time point at which a treatment was applied generating an expression response, with evidence that gene profiles could be described by a standard response function. For the *E. coli *study [21] the master regulatory gene *sox*S demonstrated an exponential-type response to the application of paraquat. To identify all genes with a similar exponential shape of response, an exponential function was fitted using the regression modelling approach to all gene expression profiles from this microarray study, and the significance of each fit was determined. Similarly, a number of genes from *R. norvegicus *liver tissue treated with corticosteroid displayed profiles appearing to follow the same critical exponential curve as fitted to the *A. bisporus *data (Equation 1) [22]. The critical exponential function was thus fitted to all genes in this dataset to determine the proportion of gene profiles that were adequately described by this function.

## Authors' contributions

DCE participated in the design of the study, prepared all samples, carried out molecular genetic studies and drafted the manuscript. AM carried out statistical analysis and helped draft the manuscript. MJS participated in the design of the study and aided in molecular genetic studies. KSB conceived of the study, participated in its design and helped to draft the manuscript.

## Supplementary Material

Additional file 1 **Northern hybridizations for all five genes in each experiment**. (A) 0–24 hr experiment, (B) 0–5 day experiment, (C) tissues over 2 day experiment. 28S rRNA = loading control, CBP = cruciform DNA-binding protein, CYP II = cytochrome P450II, GHYD = glucuronyl hydrolase, GSYN = β (1–6) glucan synthase, and RAFE = riboflavin aldehyde-forming enzyme Click here for file

Additional file 2 **Comparison of gene expression responses measured using Northern analysis and qRT-PCR: correlation coefficient**. Summary of linear regression and exponential regression fits (larger values indicate a better fit), and minimum and maximum values of gene expression as measured by qRT-PCR. (CBP = cruciform DNA-binding protein, CYP II = cytochrome P450II, GHYD = glucuronyl hydrolase, GSYN = β (1–6) glucan synthase, and RAFE = riboflavin aldehyde-forming enzyme). Click here for file

Additional file 3 **Relationships between Northern analysis response and qRT-PCR measurements**. Exponential regression curve showing the relationship between the Northern analysis response (y axis) and the qRT-PCR response (x axis) for all five genes and all three experiments. Column 1 is for the 0–24 hr experiment, column 2 is for the 0–5 day experiment, column 3 is for the tissues over 2 day experiment. Each row is for a different gene: CBP = cruciform DNA-binding protein, CYP II = cytochrome P450II, GHYD = glucuronyl hydrolase, GSYN = β (1–6) glucan synthase, and RAFE = riboflavin aldehyde-forming enzyme Click here for file
