# Supplementary material for: Statistical modelling of transcript profiles of differentially regulated genes
Source: BMC Mol Biol. 2008 Jul 23;9:66. doi: 10.1186/1471-2199-9-66 (PMC2525656; doi:10.1186/1471-2199-9-66)
Supplement: Additional file 1 — Northern hybridizations for all five genes in each experiment. (A) 0–24 hr experiment, (B) 0–5 day experiment, (C) tissues over 2 day experiment. 28S rRNA = loading control, CBP = cruciform DNA-binding protein, CYP II = cytochrome P450II, GHYD = glucuronyl hydrolase, GSYN = β (1–6) glucan synthase, and RAFE = riboflavin aldehyde-forming enzyme [file 1471-2199-9-66-S1.doc]

**Additional file 1 – Northern hybridizations for all five genes in each experiment.** (A) 0-24 hr experiment, (B) 0-5 day experiment, (C) tissues over 2 day experiment. 28S rRNA = loading control, CBP = cruciform DNA-binding protein, CYP II = cytochrome P450II, GHYD = glucuronyl hydrolase, GSYN = β (1-6) glucan synthase, and RAFE = riboflavin aldehyde-forming enzyme

1A

| Gene | Time (hours)  0 3 6 9 12 15 18 21 24 |
| --- | --- |
| 28S rRNA | 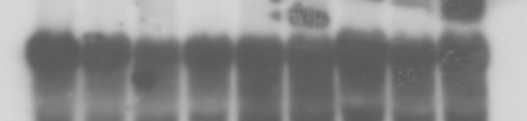 |
| CBP | 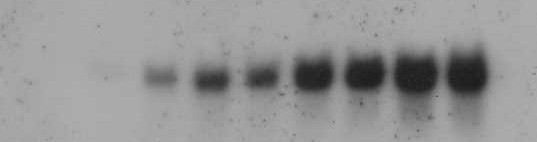 |
| CYPII | 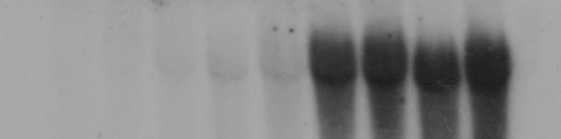 |
| GHYD | 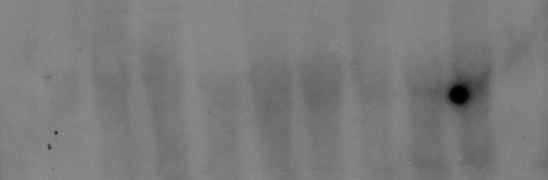 |
| GSYN | 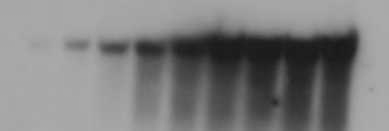 |
| RAFE | 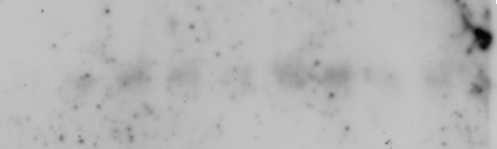 |

1B

| Gene | Time (days)  0 0 1 1 2 2 3 3 4 4 5 5 |
| --- | --- |
| 28S rRNA | 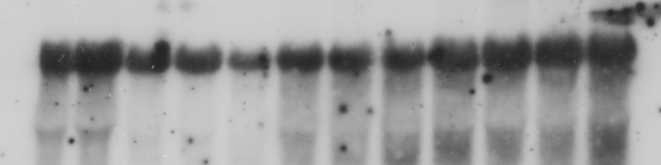 |
| CBP | 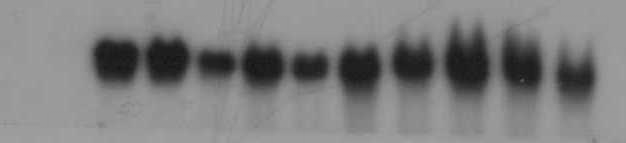 |
| CYPII | 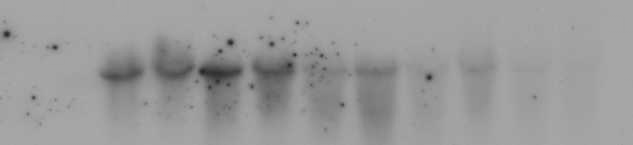 |
| GHYD | 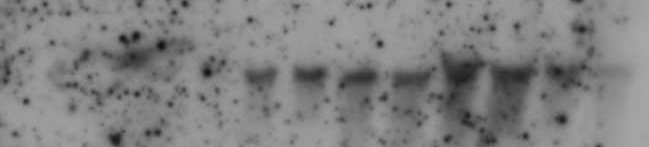 |
| GSYN | 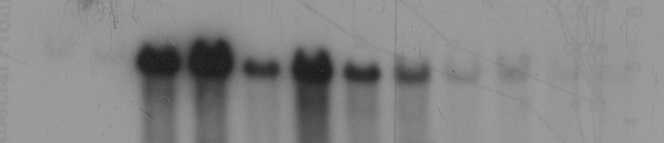 |
| RAFE | 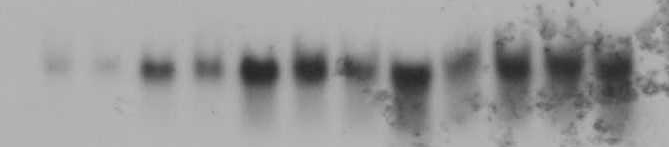 |

1C

| Gene | Stipe Cap Gills |
| --- | --- |
|  | Time (days)  0 1 2 0 1 2 0 1 2 |
| 28S rRNA | 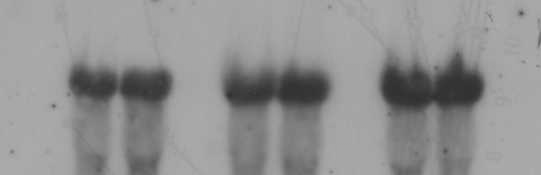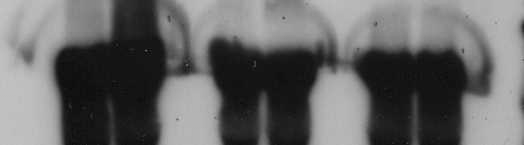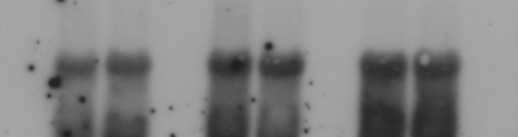 |
| CBP | 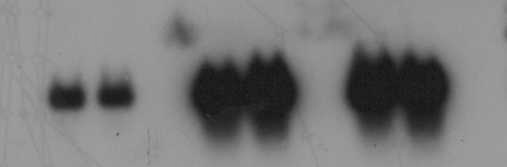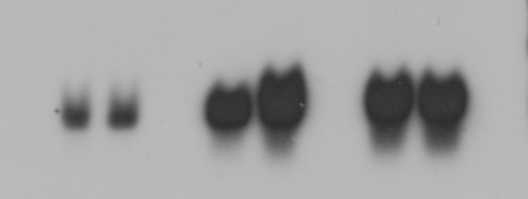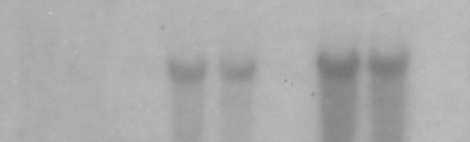 |
| CYPII | 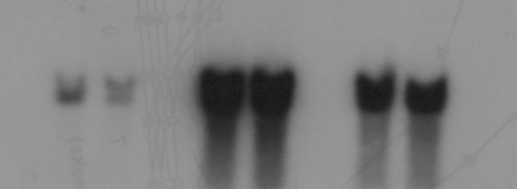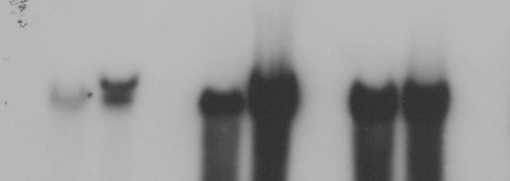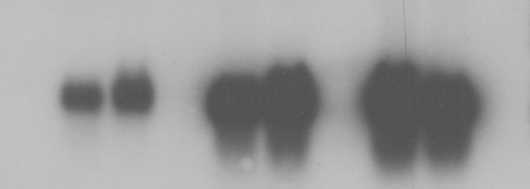 |
| GHYD | 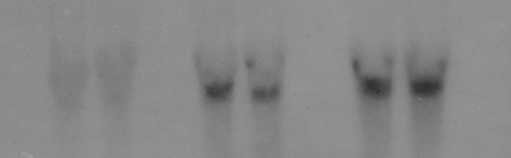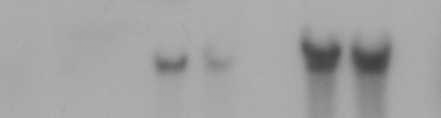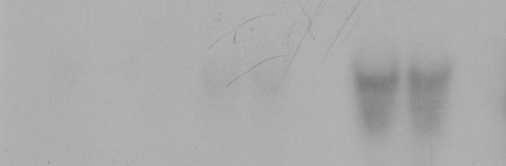 |
| GSYN | 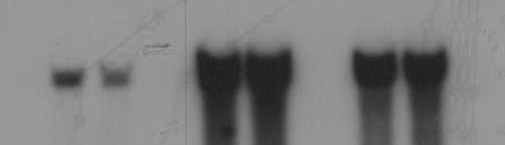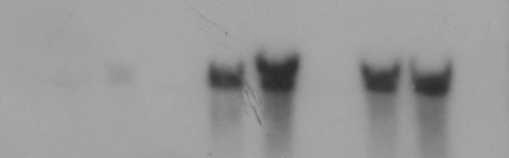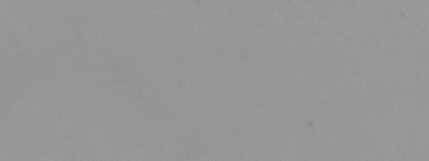 |
| RAFE | 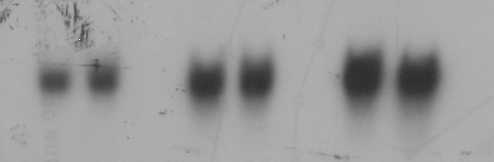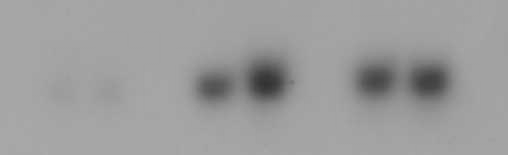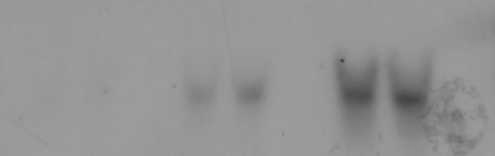 |
