# Supplementary material for: Statistical modelling of transcript profiles of differentially regulated genes
Source: BMC Mol Biol. 2008 Jul 23;9:66. doi: 10.1186/1471-2199-9-66 (PMC2525656; doi:10.1186/1471-2199-9-66)
Supplement: Additional file 2 — Comparison of gene expression responses measured using Northern analysis and qRT-PCR: correlation coefficient. Summary of linear regression and exponential regression fits (larger values indicate a better fit), and minimum and maximum values of gene expression as measured by qRT-PCR. (CBP = cruciform DNA-binding protein, CYP II = cytochrome P450II, GHYD = glucuronyl hydrolase, GSYN = β (1–6) glucan synthase, and RAFE = riboflavin aldehyde-forming enzyme). [file 1471-2199-9-66-S2.doc]

**Additional file 2: Comparison of gene expression responses measured using Northern analysis and qRT-PCR: correlation coefficient**, summary of linear regression and exponential regression fits (larger values indicate a better fit), and minimum and maximum values of gene expression as measured by qRT-PCR. (CBP = cruciform DNA-binding protein, CYP II = cytochrome P450II, GHYD = glucuronyl hydrolase, GSYN = β (1-6) glucan synthase, and RAFE = riboflavin aldehyde-forming enzyme)

|  |  | % variance accounted for | | qRT-PCR | |
| --- | --- | --- | --- | --- | --- |
| Gene | Correlation coefficient | Linear regression | Exponential regression | Min | Max |
| *Experiment: three hourly intervals for 24 hours (9 observations)* | | | | | |
| CBP  CYPII  GHYD  GSYN  RAFE | 0.930  0.866  0.906  0.963  0.120 | 84.6  71.5  79.5  91.7  - | 84.1  78.7  86.0  93.3  - | 0.3475  0.01871  0.02984  0.4423  0.3698 | 111.1  2.137  2.060  12.48  3.661 |
| *Experiment: twenty four hourly intervals for five days (12 observations)* | | | | | |
| CBP  CYPII  GHYD  GSYN  RAFE | 0.611  0.902  0.643  0.851  0.892 | 31.0  79.4  35.4  69.7  77.6 | 56.7  77.3  52.5  75.5  85.1 | 0.03665  0.00937  0.04678  0.06188  0.08603 | 151.6  2.705  4.320  17.28  7.267 |
| *Experiment: twenty four hourly intervals for two days, separate tissues (18 observations)* | | | | | |
| CBP  CYPII  GHYD  GSYN  RAFE | 0.657  0.822  0.610  0.686  0.868 | 39.6  65.6  33.2  43.8  73.7 | 35.6  72.6  59.8  40.1  90.2 | 0.1598  0.00612  0.01416  0.00616  0.04219 | 78.05  1.936  2.189  8.717  12.03 |
