# Supplementary material for: Statistical modelling of transcript profiles of differentially regulated genes
Source: BMC Mol Biol. 2008 Jul 23;9:66. doi: 10.1186/1471-2199-9-66 (PMC2525656; doi:10.1186/1471-2199-9-66)
Supplement: Additional file 3 — Relationships between Northern analysis response and qRT-PCR measurements. Exponential regression curve showing the relationship between the Northern analysis response (y axis) and the qRT-PCR response (x axis) for all five genes and all three experiments. Column 1 is for the 0–24 hr experiment, column 2 is for the 0–5 day experiment, column 3 is for the tissues over 2 day experiment. Each row is for a different gene: CBP = cruciform DNA-binding protein, CYP II = cytochrome P450II, GHYD = glucuronyl hydrolase, GSYN = β (1–6) glucan synthase, and RAFE = riboflavin aldehyde-forming enzyme [file 1471-2199-9-66-S3.doc]

**Additional file 3 – Relationships between Northern analysis response and qRT-PCR measurements**

Exponential regression curve showing the relationship between the Northern analysis response (y axis) and the qRT-PCR response (x axis) for all five genes and all three experiments. Column 1 is for the 0-24 hr experiment, column 2 is for the 0-5 day experiment, column 3 is for the tissues over 2 day experiment. Each row is for a different gene: CBP = cruciform DNA-binding protein, CYP II = cytochrome P450II, GHYD = glucuronyl hydrolase, GSYN = β (1-6) glucan synthase, and RAFE = riboflavin aldehyde-forming enzyme
